# Spatial Localization of EEG Electrodes in a TOF+CCD Camera System

**DOI:** 10.3389/fninf.2019.00021

**Published:** 2019-04-09

**Authors:** Shengyong Chen, Yu He, Huili Qiu, Xi Yan, Meng Zhao

**Affiliations:** ^1^College of Computer Science, Zhejiang University of Technology, Hangzhou, China; ^2^School of Computer Science and Engineering, Tianjin University of Technology, Tianjin, China; ^3^College of Business, Missouri State University, Missouri, TX, United States

**Keywords:** EEG, TOF camera, system calibration, point cloud, electrode localization

## Abstract

A crucial link of electroencephalograph (EEG) technology is the accurate estimation of EEG electrode positions on a specific human head, which is very useful for precise analysis of brain functions. Photogrammetry has become an effective method in this field. This study aims to propose a more reliable and efficient method which can acquire 3D information conveniently and locate the source signal accurately in real-time. The main objective is identification and 3D location of EEG electrode positions using a system consisting of CCD cameras and Time-of-Flight (TOF) cameras. To calibrate the camera group accurately, differently to the previous camera calibration approaches, a method is introduced in this report which uses the point cloud directly rather than the depth image. Experimental results indicate that the typical distance error of reconstruction in this study is 3.26 mm for real-time applications, which is much better than the widely used electromagnetic method in clinical medicine. The accuracy can be further improved to a great extent by using a high-resolution camera.

## Introduction

The electroencephalograph (EEG) technology is now widely used in clinical medicine such as epilepsy, coma, brain deaths and so on, due to its use, economy, safety, and non-invasive detection (Jeon et al., 2018). To well-use the EEG technology for analyzing the brain activities, it is important to accurately locate the position of scalp signal in the cerebral cortex (Qian and Sheng, 2011; Reis and Lochmann, 2015; Butler et al., 2016; Saha et al., 2017; Liu et al., 2018). At present, there are several kinds of EEG electrode localization methods, including (1) manual method, (2) digital radio frequency (RF) electromagnetic instrument, (3) magnetic resonance (MR), (4) ultrasonic transmission and reflection, and (5) photogrammetric method (Koessler et al., 2007). The manual method needs a relevant tool to measure the distance according to the preset sensor. This method is low in cost, but it is time-consuming and labor-consuming, and it is easy to cause errors due to manual operation (Russell et al., 2005). Electromagnetic RF digital instrumentation is currently the most widely utilized method. The principle is to locate the position of an EEG electrode through the magnetic field, and its accuracy is up to 4 mm. Of course, it is faster and more convenient than the manual method, but the disadvantage is that single point measurements are prone to mistakes, which means that to obtain accurate results the work needs to be repeated many times. Moreover, this method is strict with the overall measurement environment, requiring appropriate air humidity and temperature and no metal artifacts. Additional data conversion tools are also necessary. The specific implementation of the MR method requires an additional calibration object, which is not applicable to multi-sensor situations. The ultrasonic method is the same as the digital electromagnetic conversion method, which requires a single point measurement and consumes time and energy. One of the common disadvantages of the above methods is that the electrical signal will interfere with the weak EEG signals, which will affect the final detection results.

Compared with traditional methods, the photogrammetric method is fast, accurate, and easy to operate. From early 2000, Bauer et al. used a method to achieve the EEG electrode localization system with 12 industrial cameras, which did not specify the system settings and operating procedures (Bauer et al., 2000). Russell et al. used 11 sets of industrial cameras to locate the electrode position (Russell et al., 2005). The method is simple in operation, time-saving for operators, and there is no need for additional devices. The experimental process only takes 15–20 min, and patients are not required to participate in the subsequent data processing, which brings great convenience to patients and doctors. The working principle of this method is to calibrate the 11 cameras and obtain the three-dimensional (3D) information of each electrode with the ideas of stereo matching in computer vision. Yet, there are three shortcomings. Firstly, each electrode of the image must be manually marked, which is likely to cause artificial errors. Secondly, the system is only suitable for self-made electrode caps, not applicable to other types of electrode caps, but other traditional methods do not have this limitation. Thirdly, the system can only identify the visible electrode points. For some invisible electrode points which may be hidden in the hair, this method is useless, but electromagnetic digital method and ultrasonic method do not have this limitation (Zhang et al., 2014). The equipment is so complex that it is not easy to operate. Baysal and Sengül (2010) used only one camera to locate the electrode position, hoping to reduce costs. The working process is to move the camera along a pre-set route, taking pictures at every angle (Koessler et al., 2007). Although the cost is reduced, the patient must stay still for a long period of time, increasing the likelihood of human error and prolonging the duration of data acquisition.

Recently, there has been a great deal of interest in the development and applications of time-of-flight (TOF) depth cameras. In 2015, Yao et al. presented the full very large-scale integration (VLSI) implementation of a new high-resolution depth-sensing system on a chip (SoC) based on active infrared structured light, which estimates the 3D scene depth by matching randomized speckle patterns (Yao et al., 2015). At the same year, Golbach et al. presented a computer-vision system for seedling phenotyping that combines best of both approaches by utilizing TOF depth cameras (Golbach et al., 2016). Although TOF has its unique features, the practical applicability of TOF cameras is still limited by low resolution and quality of depth measurements. This has motivated many researchers to combine TOF cameras with other sensors in order to enhance and upsample depth images (Eichhardt et al., 2017). Calibration between depth cameras and other sensors has become a major concern. A modified method about multi-modal camera calibration is proposed in this report.

In summary, methods in previous studies, to some degree, can solve data acquisition and operability, but there are still many limitations. This report proposes a convenient and accurate method, which is also based on the photogrammetry principle (Russell et al., 2005; Clausner et al., 2017). The acquisition system of EEG signals based on RGB-Depth (RGB-D) multi-modal data is constructed by using the high resolution industrial camera and the high precision depth camera to capture the object's distance and color information simultaneously. The system captures images from five perspectives, which contains all the collected electrodes from all the perspectives. Electrode distribution of the electrode cap adopts the international 10–20 standard. The information collecting process can be performed in real-time. All image processing algorithms are achieved off-line, which greatly improves the flexibility and operability of the system.

This article reports the design of such a photogrammetry system both theoretically and experimentally. The remainder of this report is structured as follows. Section Technology and Implementation introduces the implementation technology, including the sensing method, camera calibration, and singular value decomposition (SVD) algorithm. The experimental process for electrode identification and localization will be presented in section Experiments and Results. Finally, the report summarizes the findings and concluding remarks.

## Technology and Implementation

### System Setup

The existing photogrammetric methods, whether measured through a monocular, binocular, or multi-camera system, without exception, are to obtain 3D information of the electrode positions by adopting the stereo vision method. Theoretically, each electrode point needs to be captured by two or more cameras. They need to deal with more pictures, and the algorithm is more complex. Therefore, this report proposes the use of a depth camera, MESA-SR-4000, based on TOF technology, which can directly obtain the depth information. The existing depth camera cannot directly identify the position of the EEG electrode because of its low resolution. However, the color camera can get the target color, texture and other 2D information. Hence, this project combines the two cameras to get the distance and color information of the scene. Accordingly, the EEG signal acquisition system based on RGB-D multi modal data is built. As long as all the electrodes are captured by the system, all the 3D information of the electrode can be obtained. This system can avoid the complexity of shooting the same electrode from two or more angles. Compared with the multipurpose camera, the system reduces the cost of materials, decreases the number of cameras, and greatly simplifies the algorithm. Compared with the single-camera, this system simplifies the experimental process and makes the operation simpler. There is no need to have a pre-set line nor to debug the angle of the placed mirror (Qian and Sheng, 2011), while at the same time, it improves efficiency.

The system processes in the following way. Firstly, the image is collected by using both the color camera and the depth camera. The color camera is responsible for the color picture of the electrode, so that the EEG electrode can be conveniently detected in the image and the 2D information of the electrode can be obtained. The depth camera is responsible for obtaining the point cloud data of the electrode, so that distance information of the electrode can be obtained. The key issue is the calibration of two different cameras. Secondly, this project uses the multi-camera measurement scheme, which can obtain all the electrodes, rather than the distance information. In this project, a five-camera group is applied to photograph the experimental targets in five angles. The five angles are located around the head. Of course, if the experimental equipment is not complete, the same camera group can also be located around the head at five angles, respectively. Ideally all the electrode information can be captured by the camera in five angles. Compared with the color camera based photogrammetry system, the photogrammetric system designed in this project has greatly reduced the number of angles taken and the complexity of the systematic framework.

**Figure 1:**
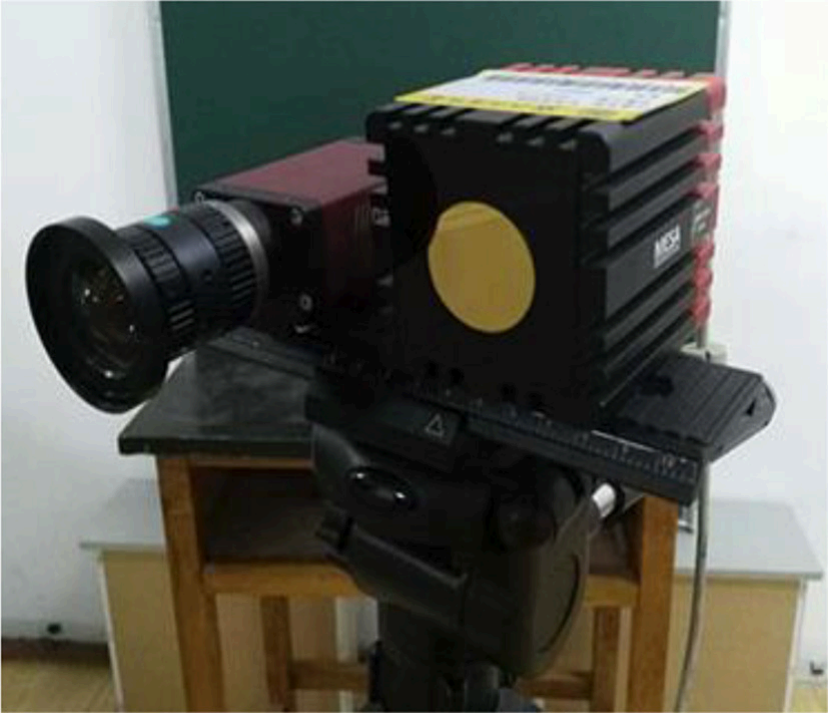
The camera system.

**Figure 2:**
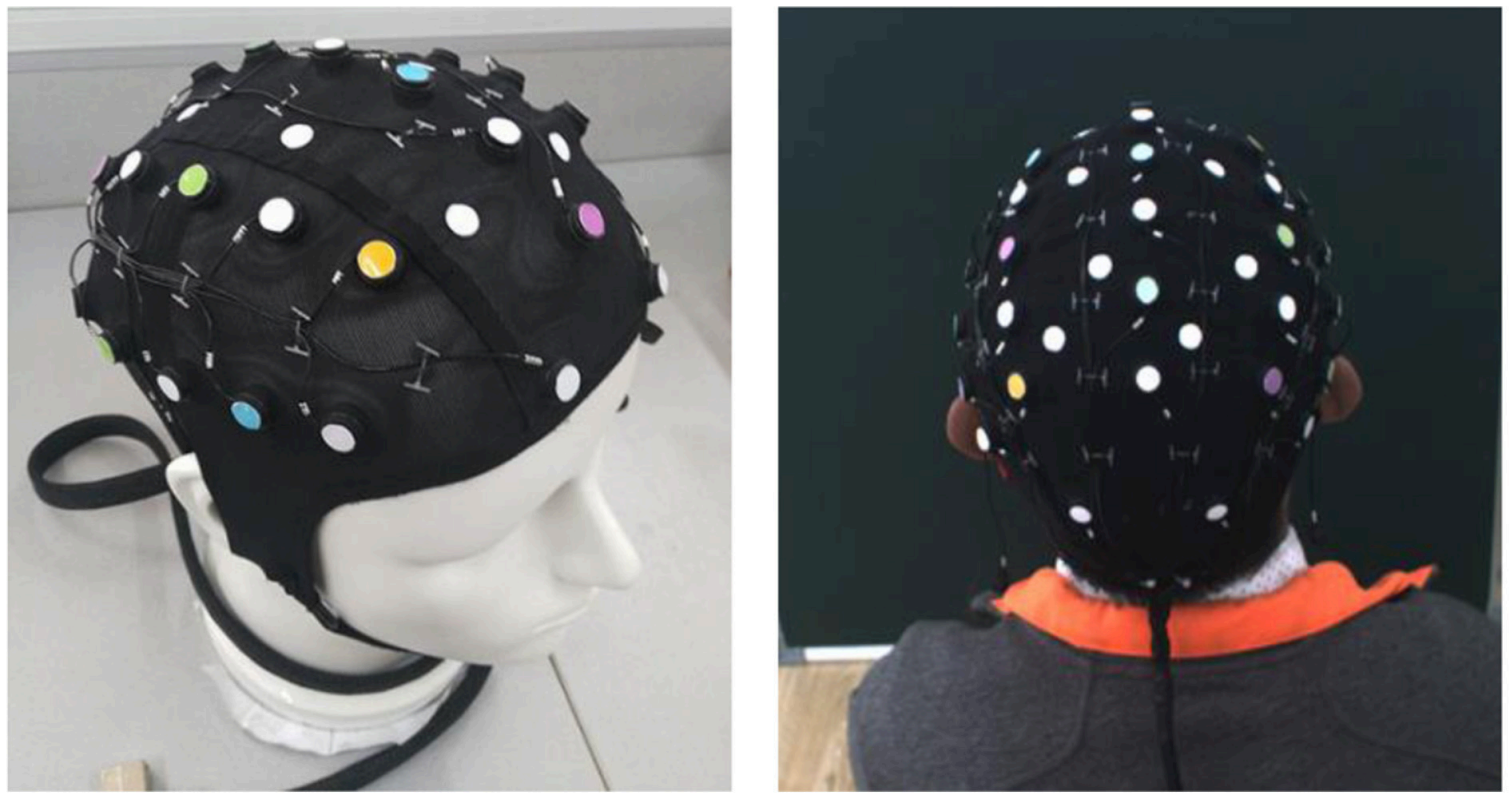
The electrode cap on a head model and on a subjective head.

In this project, the resolution of the color camera CCD is 1,624 × 1,234, and the depth camera TOF (MESA-SR-4000) has a resolution of 176 × 144. The combined camera system is shown in Figure 1. The electrode cap covered on a head model and a subjective head for practical tests are shown in Figure 2. The 10–20 electrodes are organized on a cap that is placed on the heads. The different colors on the electrode dot can easily be made, e.g., using some paint coat or sticky paper. In either way it is also easy to change colors. Making the dot colors does not affect the electrode functions or costs.

**Figure 3:**
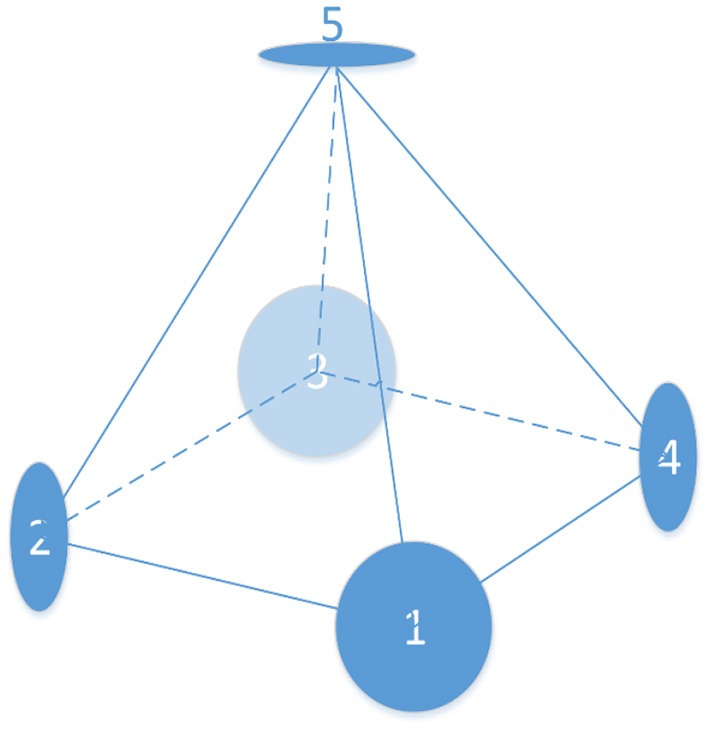
The schematic diagram.

According to the accuracy of the TOF camera's sensing range, the best shooting distance of the TOF camera is between 0.5 and 8 m. The schematic diagram is shown in Figure 3. Five groups of cameras are used in this system to take pictures simultaneously, four (1, 2, 3, and 4 in Figure 3) of which are aligned around the head with an angle of 90°, while the last is located overhead. Then all the electrodes will be reconstructed through the color image captured by the CCD camera and depth information is obtained by the TOF camera. The target RGB-D data is obtained from multiple angles. The horizontal distance between the model and the camera is 60 cm and the vertical distance is 40 cm.

**Figure 4:**
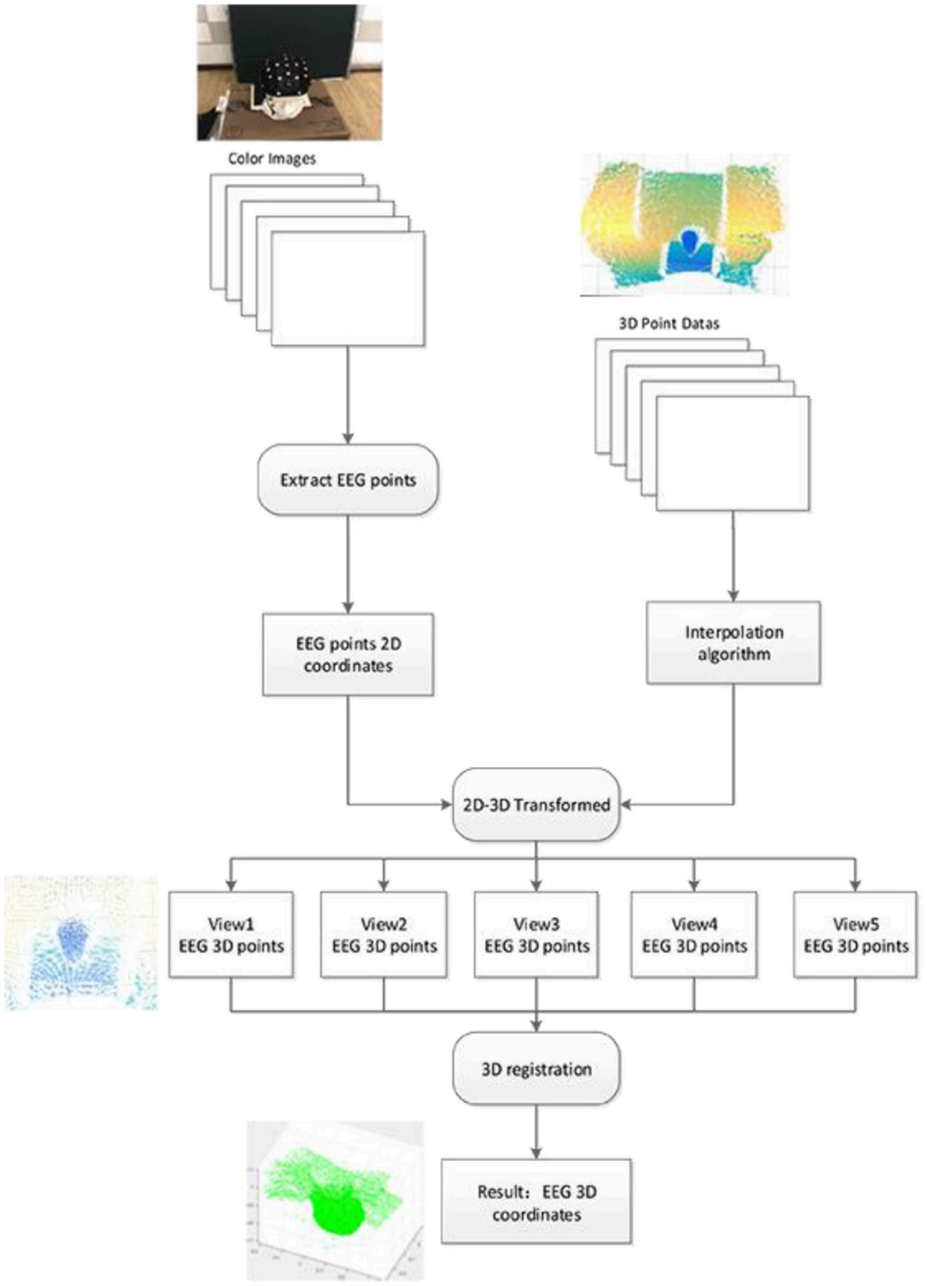
Data processing flow.

The operational flow of the system is shown in Figure 4. Firstly, the color images and the 3D point cloud data are obtained by using the color camera and the depth sensor in five angles. Then, electrode coordinates are detected and extracted in color images. Its coordinates in 3D space can be calculated by using the calibration results of color camera and depth sensor. Finally, the correlation algorithm is used to calculate the relationship between the five coordinate systems of the five views (Wang et al., 2017). Therefore, all the electrodes of different angles of view in five different coordinate systems are registered in the same spatial coordinate system.

### System Calibration

Traditionally, the calibration method utilizes the depth map obtained by the TOF camera and the color map obtained by the CCD camera to complete calibration (Cheng et al., 2016; Raposo et al., 2016). Nevertheless, the resolution of the depth image is very low, and the results are often unstable. In order to solve this problem, this project uses a new calibration plate and accurate point cloud data to perform camera calibration (Jung et al., 2015; Wei and Zhang, 2015). The comparison of the two methods will be described in the next section. The camera calibration model is designed as follows. Assuming that Q is a point in the space, the coordinates of the camera coordinate system are ${\left(x_{c},y_{c},z_{c}\right)}^{T}$. The projection of point Q in the normalized image is ***X***_n_

(1) $$\begin{array}{r} X_{n}=\left[\begin{array}{c} x\\ y \end{array}\right]=\left[\begin{array}{c} x_{c}/z_{c}\\ y_{c}/z_{c} \end{array}\right] \end{array}$$

If taking into account the lens distortion, the above coordinates are mapped ***X***_d_

(2) $$\begin{array}{r} X_{d}=\left[\begin{array}{c} x^{\prime }\\ y^{\prime } \end{array}\right]={\left(1+k_{1}r^{2}+k_{2}r^{4}\right)X}_{n} \end{array}$$

where $r=\sqrt{x^{2}+y^{2}}$, *k*_1_, *k*_2_ are radial distortion coefficients. ***X***_d_ is mapped to the image coordinates ***X***_q_, i.e.,

(3) $$\begin{array}{r} X_{q}=\left[\begin{array}{c} x^{{}^{*}}\\ y^{{}^{*}} \end{array}\right]=\left[\begin{array}{c} f_{x}x^{\prime }+c_{x}\\ f_{y}y^{\prime }+c_{y} \end{array}\right] \end{array}$$

where *f*_x_ and *f*_y_ are focal length in x and y directions, respectively, and *c*_x_ and *c*_y_ are the principle point coordinates.

The relationship between the camera groups can be described as the relationship between the coordinates of the point Q in the two camera coordinates. Assuming ***X***_cd_ is the coordinate vector of the point Q in the TOF camera coordinate system, ***X***_cc_ is the coordinate vector of the point Q in the CCD camera coordinate system, and their relationship can be described as

(4) $$\begin{array}{r} X_{cc}={RX}_{cd}+T \end{array}$$

The goal of calibration is to solve the rotation matrix *R* and the translation matrix *T*.

### Decomposition for Data Stitching

With regard to the point cloud stitching problem, many works use an ICP algorithm or improved ICP algorithm (Cheng et al., 2016; Yang et al., 2016). However, here, due to the large deviation of the angle of view, the performance of ICP algorithm is not ideal, thus SVD is adopted to calculate the conversion relationship between the two sets of point clouds (Sorkine, 2009; Jung et al., 2015; Raposo et al., 2016). The principle is described firstly from this transform

(5) $$\begin{array}{r} \left(R,t\right)=\text{armgin}\overset{n}{\underset{i=1}{\sum }}w_{i}||\left(Rm_{i}+t\right)-n_{i}|{|}^{2} \end{array}$$

where *w*_i_ >0 is the weight of each point in the cloud. Calculate the displacement, and the above formula *R* is set to invariant to derive *t*, at the same time *F*_(t)_ = (*R, t*), which has the derived derivative

(6) $$\begin{array}{r} 0=\frac{\partial F}{\partial t}=\sum_{i=1}^{n}2w_{i}\left(Rm_{i}+t-n_{i}\right)\\ =2t\left(\sum_{i=1}^{n}w_{i}\right)+2R\left(\sum_{i=1}^{n}w_{i}m_{i}\right)\;-2\sum_{i=1}^{n}w_{i}n_{i} \end{array}$$

where

(7) $$\begin{array}{r} \bar{m}=\frac{\sum_{i=1}^{n}w_{i}m_{i}}{\sum_{i=1}^{n}w_{i}},\bar{n}=\frac{\sum_{i=1}^{n}w_{i}n_{i}}{\sum_{i=1}^{n}w_{i}} \end{array}$$

(8) $$\begin{array}{r} \text{t}=\bar{n}-R\bar{m} \end{array}$$

Substitute (6–8) into (5) and we have

(9) $$\begin{array}{l} \sum_{i=1}^{n}w_{i}{\left\|\left(Rm_{i}+t\right)-n_{i}\right\|}^{2}=\sum_{i=1}^{n}w_{i}{\left\|Rm_{i}+\bar{\;n}-R\bar{\;m}-n_{i}\right\|}^{2}\\ \;=\sum_{i=1}^{n}w_{i}{\left\|R(m_{i}-\bar{m})-(n_{i}-\bar{\;n})\right\|}^{2} \end{array}$$

(10) $$\begin{array}{r} X_{i}:=m_{i}-\bar{\;m},\;Y_{i}:=n_{i}-\bar{\;n}. \end{array}$$

(11) $$\begin{array}{r} \text{R}=\text{armgin}\overset{n}{\underset{i=1}{\sum }}w_{i}||RX_{i}-Y_{i}|{|}^{2} \end{array}$$

To calculate the amount of rotation (11), is expanded in a matrix representation,

(12) $$\begin{array}{r} ||RX_{i}-Y_{i}|{|}^{2}={\left(RX_{i}-Y_{i}\right)}^{T}\left(RX_{i}-Y_{i}\right)\\ \;=\left({X_{i}}^{T}{\text{R}}^{T}-{Y_{i}}^{T}\right)\left(RX_{i}-Y_{i}\right)\\ ={X_{i}}^{T}{\text{R}}^{T}RX_{i}-{Y_{i}}^{T}RX_{i}-{X_{i}}^{T}{\text{R}}^{T}Y_{i}+{Y_{i}}^{T}Y_{i}\\ ={X_{i}}^{T}X_{i}-{Y_{i}}^{T}RX_{i}-{X_{i}}^{T}{\text{R}}^{T}Y_{i}+{Y_{i}}^{T}Y_{i} \end{array}$$

Since the rotation matrix R is an orthogonal matrix, there is *R*^*T*^*R* = 1. ${Y_{i}}^{T}RX_{i}$ and ${X_{i}}^{T}{\text{R}}^{T}Y_{i}$ are scalar. The transposition of the scalar is still equal to the scalar itself, i.e.,

(13) $$\begin{array}{r} {X_{i}}^{T}{\text{R}}^{T}Y_{i}={\left({X_{i}}^{T}{\text{R}}^{T}Y_{i}\right)}^{T}={Y_{i}}^{T}RX_{i}. \end{array}$$

(14) $$\begin{array}{r} ||RX_{i}-Y_{i}|{|}^{2}={X_{i}}^{T}X_{i}-{2Y_{i}}^{T}RX_{i}+{Y_{i}}^{T}Y_{i} \end{array}$$

Only one of them is related to R and transforms it into the minimum of its variable,

(15) $$\begin{array}{r} \text{armgin}\left(-2\overset{n}{\underset{i=1}{\sum }}w_{i}{Y_{i}}^{T}RX_{i}\right) \end{array}$$

(16) $$\begin{array}{r} \text{armgin}\left(-2\overset{n}{\underset{i=1}{\sum }}w_{i}{Y_{i}}^{T}RX_{i}\right)=\text{armgin}\overset{n}{\underset{i=1}{\sum }}w_{i}{Y_{i}}^{T}RX_{i}. \end{array}$$

(17) $$\begin{array}{r} \overset{n}{\underset{i=1}{\sum }}w_{i}{Y_{i}}^{T}RX_{i}=tr\left(WY^{T}RX\right) \end{array}$$

The conversion of the above formula makes a switch from cumulative to matrix based multiplication. Here, **W** is a diagonal matrix of n × n, and **X** and **Y** are 3 × n matrices. The traces of these matrices are equal to the left-hand side of the equation.

(18) $$\begin{array}{r} \text{tr}\left(\text{AB}\right)=\text{tr}\left(\text{BA}\right) \end{array}$$

(19) $$\begin{array}{r} \text{tr}\left(WY^{T}RX\right)=\text{tr}\left(\left(WY^{T}\right)\left(RX\right)\right)=\text{tr}\left(RXWY^{T}\right) \end{array}$$

(20) $$\begin{array}{r} S=XWY^{T},svd\left(S\right)\to S=U\sum V^{T} \end{array}$$

(21) $$\begin{array}{r} \text{tr}\left(RXWY^{T}\right)=\text{tr}\left(\text{RS}\right)=\text{tr}\left(RU\sum V^{T}\right)=\text{tr}\left(\sum V^{T}RU\right) \end{array}$$

The last step of the above transformation also uses the nature of (18). Since U, R, and V are orthogonal matrices, *O* = *V*^*T*^*RU* is also an orthogonal matrix.

(22) $$\begin{array}{r} I={\text{o}}_{j}^{T}{\text{o}}_{j}=\overset{d}{\underset{i=1}{\sum }}{\text{o}}_{ij}^{2}\Rightarrow {\text{o}}_{ij}\leq 1\Rightarrow |{\text{o}}_{ij}|<1 \end{array}$$

(23) $$\begin{array}{r} \text{tr}\left(\sum O\right)=\left(\begin{array}{ccc} \sigma_{1} & \cdots & 0\\ \vdots & \ddots & \vdots \\ 0 & \cdots & \sigma_{d} \end{array}\right)\left(\begin{array}{cccc} o_{11} & o_{12} & \ldots & o_{1d}\\ o_{21} & o_{22} & \ldots & o_{2d}\\ \vdots & \vdots & \vdots & \vdots \\ o_{d1} & o_{d2} & \ldots & o_{dd} \end{array}\right)\\ =\overset{d}{\underset{i=1}{\sum }}\sigma_{i}o_{ii}\leq \overset{d}{\underset{i=1}{\sum }}\sigma_{i} \end{array}$$

From the above two terms, if the maximum trace is required, we must make the value of *O*_*ii*_ equal to I, while O is the orthogonal matrix. So, O must be the unit matrix

(24) $$\begin{array}{r} \text{I}=\text{O}=V^{T}RU\Rightarrow V=RU\Rightarrow R=VU^{T} \end{array}$$

## Experiments and Results

This section contains two parts, i.e., camera calibration and electrode identification and localization. The accuracy of camera calibration plays a very important role in the whole system. In this part, a new calibration method for the depth camera is proposed and compared with the traditional method. The experimental results show that the accuracy of our calibration method is more significant. The experimental procedure of electrode identification and localization is also described in detail in this part.

### Calibration

The traditional method to calibrate the TOF camera and the CCD camera (Wei and Zhang, 2015; Bonnabel et al., 2016; Onunwor and Reichel, 2017) produces very unsatisfactory results because the resolution of the TOF camera is quite different from the CCD camera resolution, and the acquired parameters are very unstable. The pixel of the depth image acquired by the depth camera represents the distance from the subject to the camera. In 2012, Li and Zhuo proposed a 2.5D calibration plate that takes full advantage of the depth image characteristics, which improves the accuracy of camera registration, and simplifies the complexity of the algorithm (Li and Zhuo, 2012).

**Figure 5:**
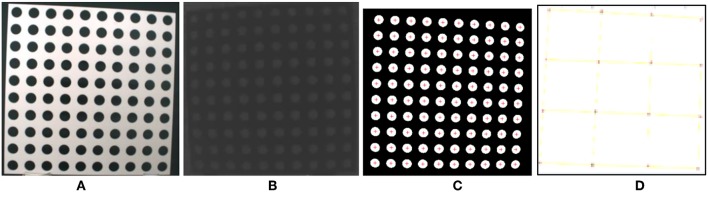
Calibration plate. **(A)** Color image of the target, **(B)** depth image, **(C)** detected points, **(D)** fitted points.

Figure 5 shows the calibration plate designed in this project. Figure 5A is the color image of the calibration plate. Figure 5B shows the depth image of the calibration plate. The size of the calibration plate is 500 × 500 mm, round hole diameter is 30 mm, and pitch of holes is 50 mm, there are 100 holes. The characteristic point is the center of each circular hole of the calibration plate.

The calibration process has two main steps. The first is to extract calibration points, i.e., to select a region of interest (ROI), to binarize the image by an automatic threshold, to remove image noise, to calculate the connected area, and to determine the center of each connected area, as shown in Figure 5C. The center of the connected area is regarded as a feature point. The second step is to fit feature points. The least square method is used to fit the characteristic points of each column and row in order to reduce the position error, as shown in Figure 5D.

**Figure 6:**
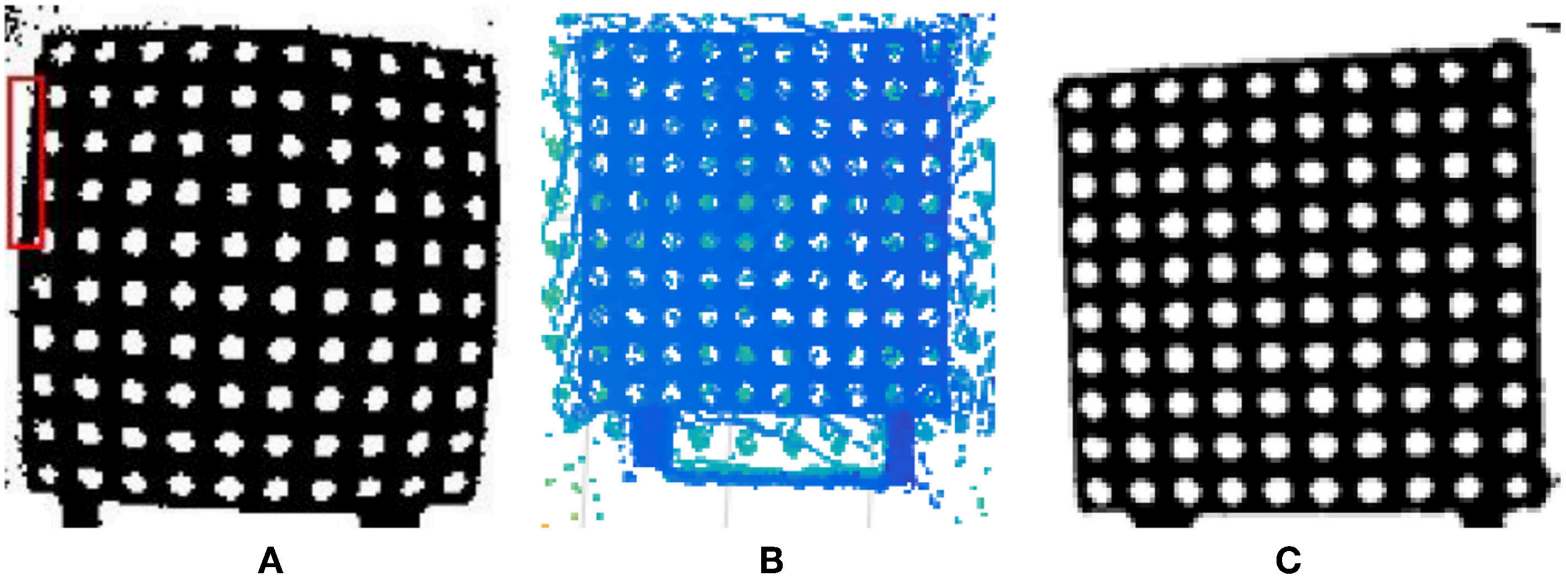
Comparison of the depth map and point cloud. **(A)** Original depth map, **(B)** point cloud, **(C)** projected coordinates without distortion.

The above method improves the accuracy of registration, yet the depth map still has radial distortion, as shown in Figure 6A. Although the use of fitting feature points can reduce errors, there is still room for improvement. Therefore, this report modifies the process of cameral calibration proposed in Li and Zhuo (2012) by employing accurate point cloud data other than the depth map. The specific process in this report includes two stages. The first is point cloud interpolation. Since the TOF camera has a low resolution, in order to obtain more accurate data, the system uses the bilinear interpolation algorithm to interpolate the point cloud data, so that its resolution is consistent with the color map. The second stage is to convert a point cloud to a 2D image. Since the point cloud represents 3D data, it cannot be directly calibrated with the color image, and thus the point cloud is required to be converted into a 2D image. In this project, the 3D coordinates are projected onto the 2D plane using the pinhole model as the theoretical basis. The result is indicated in Figures 6B,C. Compared with Figures 6A,C, we may discover that the image distortion is almost resolved.

**Figure 7:**
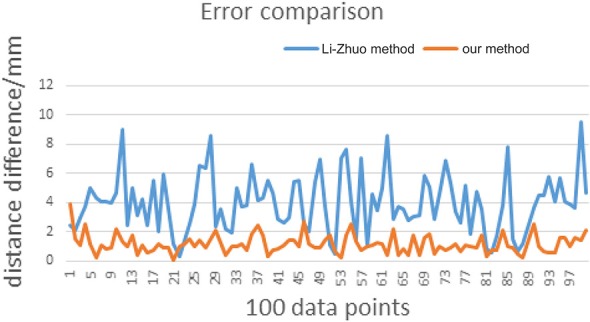
Error comparison.

According to the results obtained by the two methods, we can compare the distance errors of the two sets of points. The abscissa represents 100 data points, and the ordinate represents the distance difference between the two points before and after the calibration. Figure 7 shows the comparison of errors caused by Li-Zhuo method (Li and Zhuo, 2012) and the proposed method in this study. From the data we can find that the calibration error has dropped from the original average 3.95–1.16 mm.

### Electrode Point Identification and Localization

#### Electrode Identification

**Figure 8:**
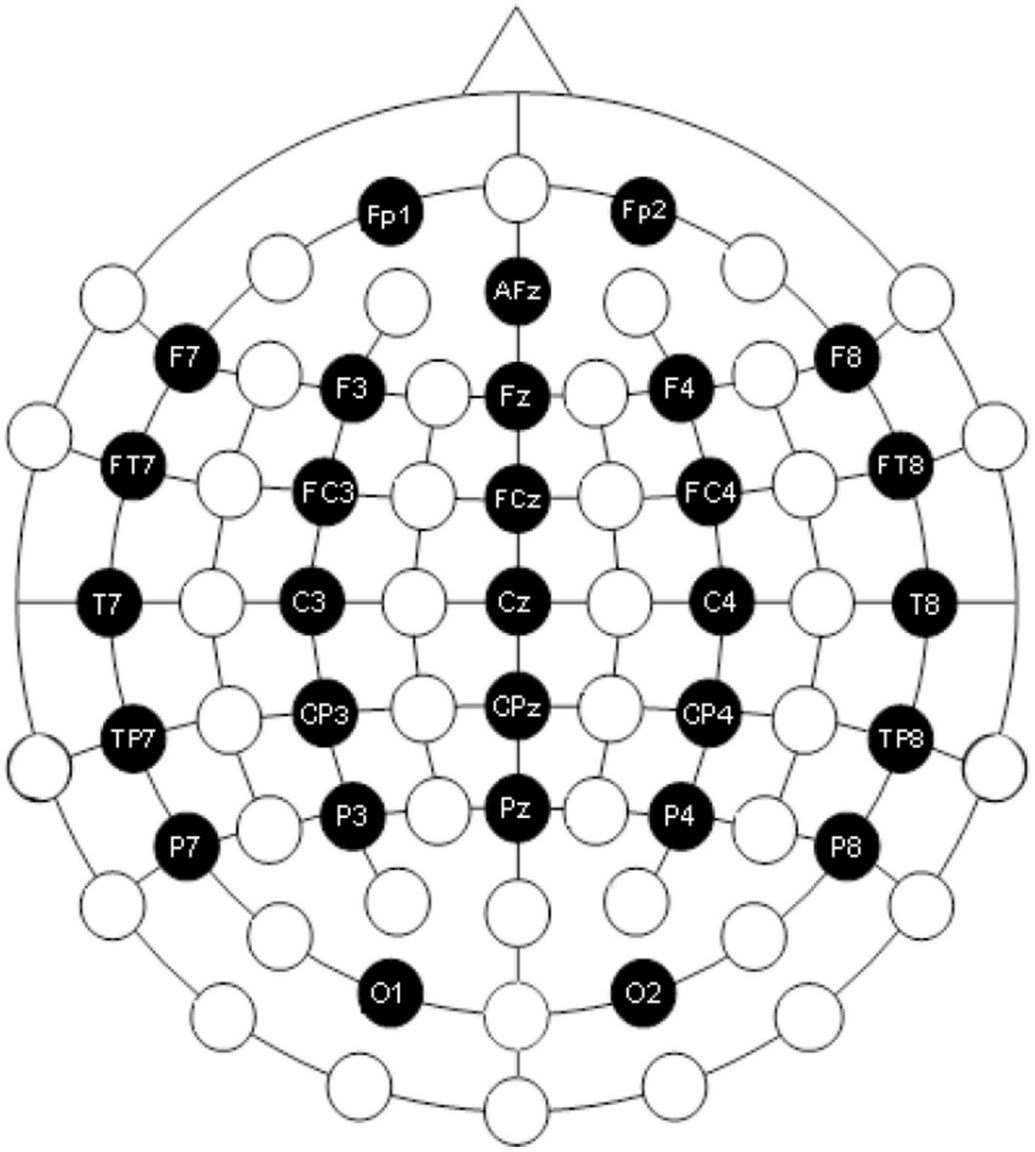
Electrode distribution diagram.

Assume that the electrode cap has a 30-channel EEG amplified signal recorder (Trotta et al., 2018). The electrode dot distribution diagram, provided by the electrode cap manufacturers, is typically shown in Figure 8. The electrodes here are marked with black color, and those names are shown in the figure.

**Figure 9:**
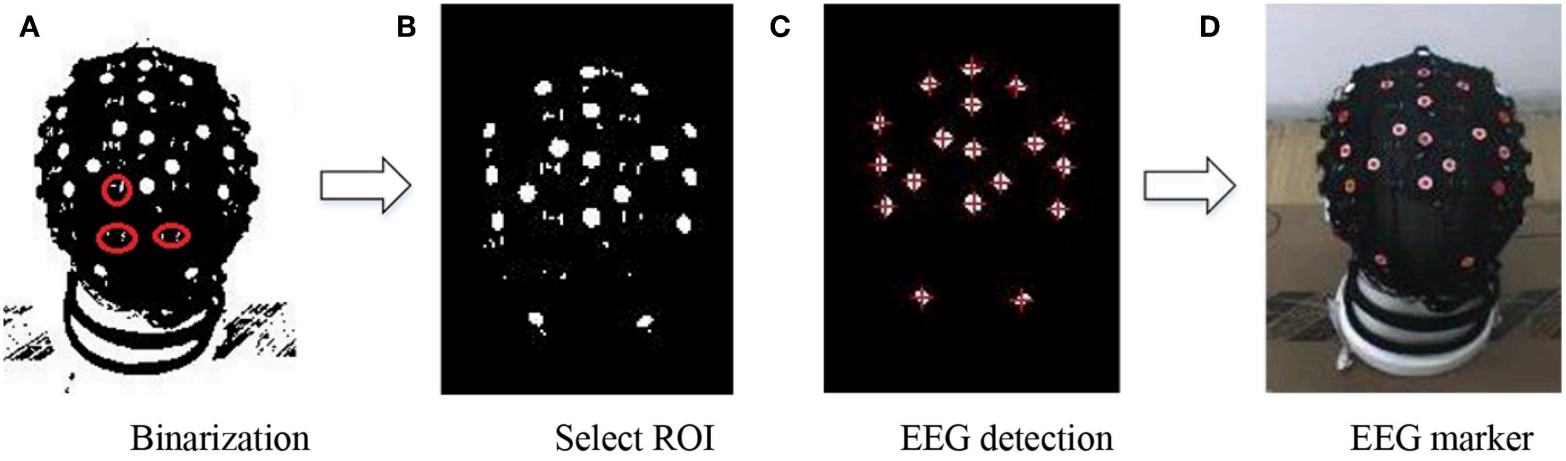
Real electrode detection on the EEG signal cap **(A)** Binarization **(B)** Select ROI **(C)** EEG detection **(D)** EEG marker.

If the precise position of the EEG electrode in color image is determined, the 3D location of the EEG electrode can be calculated by the transformation presented in section Calibration, using the similar calibration equations. In order to get precise EEG electrodes in the color image, this project adopts a method by detecting the connected region of the color image. When the electrode is detected in the color image, there will be a lot of interference because of the real electrode cap. When the color image is binarized with the appropriate threshold, there are lots of little interference regions, as shown in Figure 9A. In order to solve this problem, all connected regions are calculated and labeled, and the area of each connected region is calculated, after selecting the ROI, which contains the electrodes on the head in this picture, as shown in Figure 9B. In order to detect the electrode accurately, the algorithm adaptively adjusts the appropriate area threshold to preserve the connected area larger than the threshold, filter out the connected area less than the threshold, as shown in Figure 9C. This method reduces the noise of the electrodes. Then, the center of the connected region is calculated, and that is the center of the electrode. The coordinates of the center point are used as the positions of the electrodes, as shown in Figure 9D. Taken as an example, Figure 9 shows the image of view 3, and other views have the same process. When this step is finished, there are five color images with detected electrodes.

#### Electrode Localization

**Figure 10:**
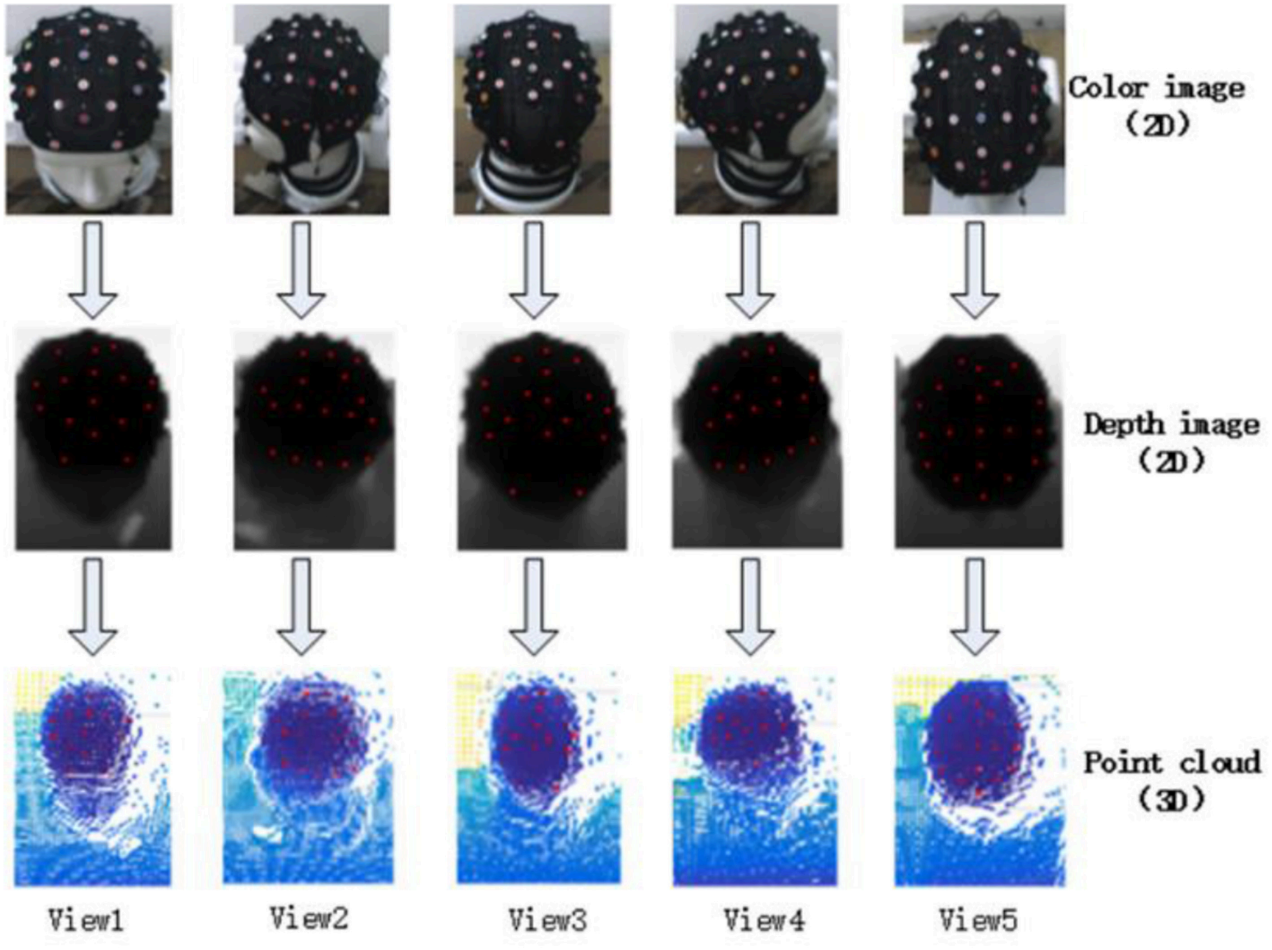
EEG electrode registration.

Figure 10 shows the five shot images from each direction. The first row is the color image which is obtained by the CCD camera, with the detected electrodes. The second row is the depth image which is obtained by the TOF camera with the transformational electrodes, and the third row is the point cloud data with the 3D electrode positions. The electrodes in the first row are detected by the method described in section Electrode identification. The electrodes in the second and third row are determined by using the transformation between the CCD camera and the TOF camera. Of course, the electrodes in the third row have three dimensions.

#### 3D Registration

**Figure 11:**
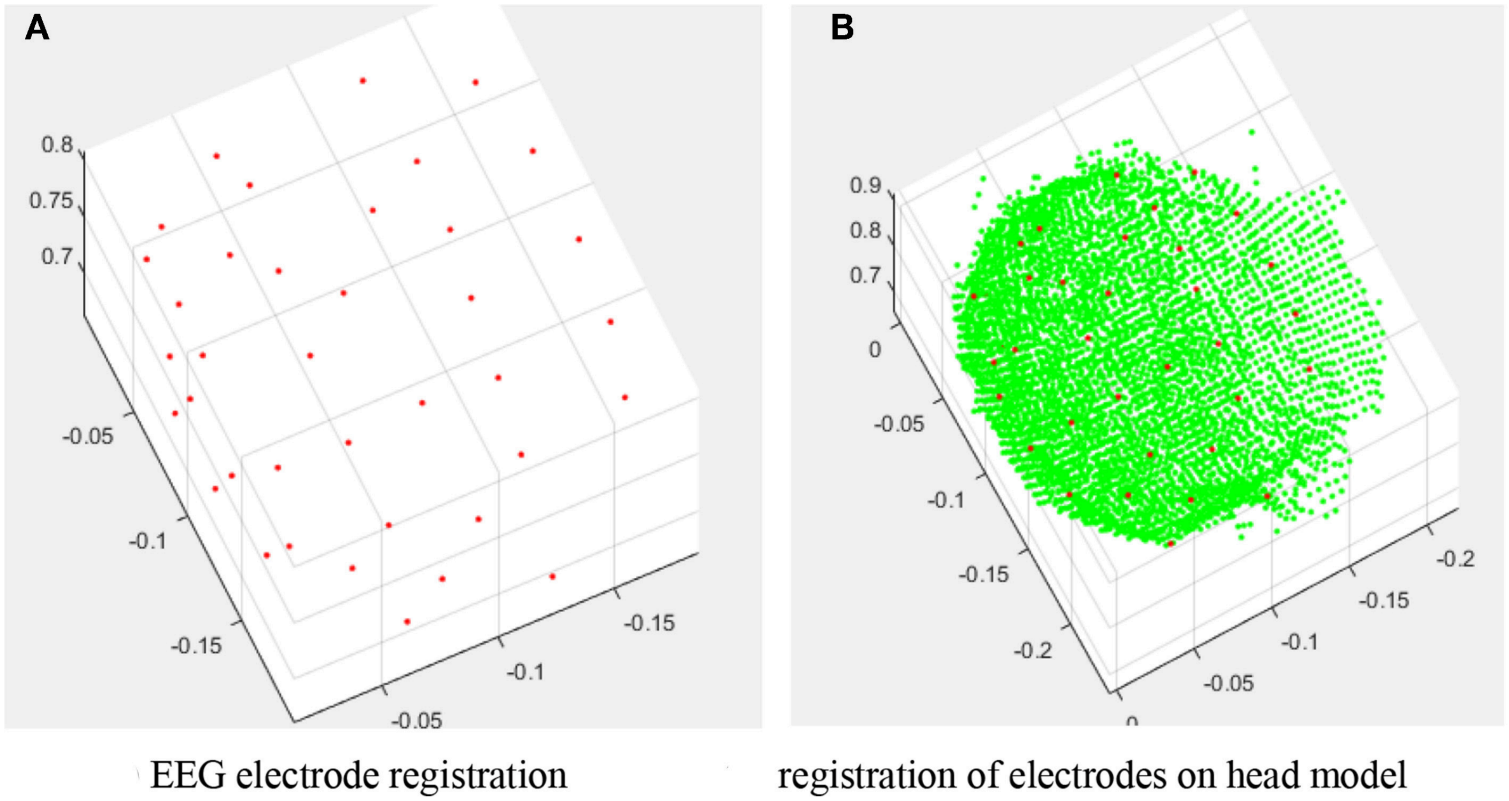
Registration results. **(A)** EEG electrode registration. **(B)** Registration of electrodes on head model.

We need to rebuild the entire brain model and the position of electrodes. In the process of point cloud stitching, many people use the classic ICP algorithm (Kim, 2015), which is only suitable for small angle stitching, i.e., with a large overlapped area, and so it is not ideal for the situation in this study. Since the angle intervals between five camera groups are relatively large, in order to reduce registration errors, this report takes the surrounding four point cloud points, i.e., view1, view2, view3, and view4, to match the view5 point cloud, respectively. The SVD algorithm described earlier in this report is used to solve the transformation relation. The electrodes, the camera angle, and the number of angles are shown in Table 1, which illustrates the situation of how the electrodes are taken. Figure 11 shows the results of the registration for all electrodes into the same coordinate system. Figure 11A is a registration diagram containing only the electrodes. Figure 11B shows the distribution of the electrodes on the head model.

**Table 1:** Electrodes, camera views, and quantities.

**Electrode**	**Camera view**	**N**
Fp1	1	1
Fp2	1	1
AFz	1 + 5	2
F8	4	1
F4	1 + 4 + 5	3
Fz	1 + 5	2
F3	1 + 2 + 5	3
F7	2	1
FT7	2	1
FC3	1 + 2 + 5	3
FCz	1 + 2 + 3 + 4 + 5	5
FC4	1 + 4 + 5	3
FT8	4	1
T8	4	1
C4	4 + 5	2
Cz	1 + 2 + 3 + 4 + 5	5
C3	2 + 5	2
T7	2	1
TP7	2	1
CP3	2 + 3	2
CPz	2 + 3 + 5	3
CP4	3 + 4	2
TP8	4	1
P7	2	1
P3	2 + 3	2
Pz	3 + 5	2
P4	3 + 4	2
P8	4	1
O1	3	1
O2	3	1

The SVD algorithm can obtain stable and reliable results with only two angles of 5–15 sets of matching points, which is much simpler than traditional photography methods. This is mainly because the TOF camera can directly obtain the exact depth of the value. In the traditional photographic methods, for either multi-camera or single-camera with multi-angle, each electrode must be shot from different directions and the depth information can be calculated according to that. The process is not only complex, but also very easy to cause human errors and matching errors. The algorithm of the multi-purpose camera method is too complicated and requires manual participation in the electrode marking process and can only use the matching electrode cap. A single camera method is a brilliant approach, yet the operation requirements are high, which is easy to cause human error. Qian and Sheng (2011) also proved that only six electrodes could reduce error when shot by more cameras, and that the other electrodes did not have this trend.

## Results

In the EEG positioning system, the inaccurate location of the electrode may cause an incorrect location of the source, and thus the accuracy of the electrode positioning is very important for research in brain science. The standard positioning error is given by Δ $=\sqrt{{\left(X_{a}-X\right)}^{2}+{\left(Y_{a}-Y\right)}^{2}+{\left(Z_{a}-Z\right)}^{2}}$, where *X, Y, Z* are estimated 3D coordinates, *X*_a_, *Y*_a_, *Z*_a_ are the real coordinate values obtained by a higher precision device, for which in this study we use a portable 3D handheld scanner, the Artec 3Ds Space Spider, with an accuracy of 0.05 mm.

The experimental process is repeated five times, using the electrode cap on the head model. A typical result of the average error of the 30 electrodes is shown in Table 2. We also tested the same process with the electrode cap on human heads and got the similar results. Therefore, with the RGB-D multi modal system, the proposed method yields an average of 3.26 mm localization error, much better than other digitizer methods where the typical equipment has a mean error of 6.1 mm. Furthermore, if we use a high-precision CCD camera for calibration and measurement, the accuracy can be easily improved up to 10 times, i.e., the error can be reduced to about 0.3 mm. Since the error is much less than the size of an electrode dot which has a diameter of 10 mm, our result is good enough for practical applications. Anyway, there are two sources of the experimental errors. One of them is the error resulting from the camera calibration, introduced in section Calibration the other is from the point cloud splicing. The points with large errors are mainly located in the edge position of the electrode cap. The error of the point in the middle position is much smaller. In fact, it is normally accepted for users if the error is <5 mm for dense arrays of electrodes. Therefore, the proposed system with this accuracy is rather sufficient for most practical applications. Some technology information and data sets carried out in this project are available on the web, http://www.sychen.com/research/vision/LEEG.htm, where some MATLAB codes are provided to demonstrate the main algorithms.

**Table 2:** Electrode positioning error (mm).

**Electrode**	**ΔX**	**ΔY**	**ΔZ**	**Δr**
Fp1	1.41	0.21	−0.37	1.47
Fp2	−1.39	−0.20	0.39	1.45
AFz	−0.79	−1.55	−2.94	3.42
F8	−4.75	0.59	−0.31	4.80
F4	−3.27	−0.88	−1.77	3.82
Fz	0.78	−0.80	2.53	2.77
F3	−2.00	2.99	−0.10	3.60
F7	2.60	−1.46	1.84	3.50
FT7	1.55	0.85	−1.27	2.18
FC3	−0.24	0.92	−1.43	1.72
FCz	1.84	−1.17	1.33	2.55
FC4	1.78	2.91	0.11	3.41
FT8	−1.15	−5.22	−0.42	5.36
T8	0.64	2.42	−2.01	3.21
C4	−3.46	−1.78	2.04	4.39
Cz	1.16	2.1	3.12	3.94
C3	−0.54	0.35	−1.32	1.47
T7	0.19	1.10	−4.01	4.16
TP7	−3.14	0.44	−0.76	3.26
CP3	1.48	−2.49	−0.56	2.95
CPz	−1.68	1.40	−1.10	2.45
CP4	3.19	1.49	0.15	3.52
TP8	0.47	2.15	0.67	2.3
P7	−1.20	−0.92	4.19	4.45
P3	1.30	−1.78	3.42	4.07
Pz	−1.31	0.01	−2.95	3.23
P4	1.76	−1.74	−0.53	2.53
P8	4.80	0.05	2.07	5.23
O1	2.89	1.13	−1.56	3.47
O2	−2.78	−1.03	1.22	3.20
AVE				3.26

There is another advantage that the method achieves good performance in terms of flexibility and simplicity of operation, which can be used in EEG source localization applications on the human brain. On the other hand, since the calibration process and brain model building can be done off-line, the on-line process only needs to detect the electrodes and map them to the brain model. This process is performed very fast and can be easily implemented for real-time applications.

## Discussion

In this study, we combine a TOF depth camera and a CCD color camera to locate the EEG electrode positions in 3D space and yield satisfactory results for practical use. Compared with the existing contributions in the literature (Table 3), such 3D positions are normally obtained by a stereo vision system, where a pair of CCD cameras used as two eyes for identification and 3-D reconstruction of electrodes. However, stereo vision is normal useful for robots but it always has its own limitations and it's still used for industrial applications, especially when there is a high requirement on precision and reliability. For example, the work by Schulze et al. (2014) is a typical realization of this technology. There are some comparisons between photogrammetry system and manual measurements or electromagnetic digitizers made in Koessler et al. (2007). One main problem of stereo vision is its reliability. The passive vision system is very sensitive to environmental conditions. When anything, like the lighting, the object size in the working space, the vision system structure, the working distance, changes, the vision system will meet a big problem of 3D reconstruction. It even could not obtain a good image for analysis anymore. The calibration of stereo cameras is very tedious because it requires an inconvenient process by an expert in robot vision. Furthermore, such a process has to be redone when either one of the settings, such as the focus, the baseline distance, the camera pose, is changed. That means such an expert has to stay there for making the system use in practical clinical applications.

**Table 3:** Comparison of the typical methods.

**Method**	**Principle**	**Equip size**	**Time**	**Accuracy**	**Reliability**	**Typical Ref**.
Manual measurement	Coordinate measuring, calipers	Small	Very slow (>10 min)	0.4 mm	Mid	De Munck et al. (1991)
Camera matrix	Stereo vision	Large	Real-time (< 0.1 s)	1.27 mm	Bad	Koessler et al. (2007)
Positioning tool	Electromagnetic digitizer	Small	5 min	2–8 mm	Mid	Dalal et al. (2014)
Photogrammetry	Structure-from-motion	Small	Slow (5–10 min)	0.8 mm	Mid	Clausner et al. (2017)
Laser scanner	Laser	Small	Slow	0.05–0.2 mm	Good	Jeon et al. (2018)
Color+depth	Color+TOF	Small	Real-time	0.3–3.3 mm	Good	This report

Regarding the locating accuracy, an error below 5–10 mm can satisfy the current EEG signal research or clinical applications. A manual process with a tool can get the accuracy of 3.6 mm, but it takes about 8 min. Schulze et al. reports their system of camera matrix can achieve a localization error of 0.761 mm. In fact, Koessler et al. (2007) already achieve the position error under 1.27 mm 10 years ago, where they distribute 11 CCD cameras on the dome for imaging. Actually, with the currently new CCD cameras, higher accuracy, e.g., 0.1 mm, can also be theoretically achieved. However, it is hard to produce general systems using such technology of stereo vision for the clinical applications. On the other hand, using laser-based equipment can, of course, get very high accuracy, e.g., the 3D handheld scanner in our laboratory can give us the accuracy of 0.05 mm.

Since there is no complicated computation required to perform the algorithms of this study, the system can be implemented for real-time applications with common personal computers. In the experiments, we mostly use ordinary devices, e.g., TOF camera (MESA-SR-4000) and CCD camera (Manta G-201C 30fps). It is performed in a personal computer with Intel i3-4130 CPU at 3.4 GHz, 4.0 GB RAM, and x64-based Windows 7 OS. A relatively lower configuration of the computer does not much affect the efficiency. Due to the resolution limited by MESA-SR-4000 and MG-201C, the result is got with a precision of 3.26 mm within 30 ms. This is usually adequate for practical real-time applications. Of course, using latest better hardware with higher resolutions, e.g., TOF camera (OPNOUS GC4 NIR) and CCD camera (Kodak KAI-08050 PoE) in our lab, we can get a corresponding higher precision but lower efficiency. Increasing the resolution of the cameras would significantly improve the accuracy, but at the same time it correspondingly decreases the efficiency. On the contrary, the number of EEG sensors has little sense to affect the performance because there are only tens of points in total.

Anyway, we have to concern the aspects of reliability, flexibility, and real-time computation for the positioning system. As we know, due to the corresponding process in stereo vision, it takes several minutes for computing and thus cannot be used for real-time purpose, e.g., when the subject needs to move the heads during a test. One advantage of the technology in this study is that it avoids the complicated computation of correspondence among multiple images, which is unlikely realized in real-time for high-resolution images on a common computer. The data acquisition and registration process is very fast by the method in this report. It means the method can be used for dynamic tests where the patient is free to move its head or body during the acquisition time. Therefore, some other research or test tasks can also be done with a system by this technology. Our method also takes advantage of flexibility. We do not need to setup a large equipment structure or working space, like a dome. The subject will also feel comfortable in the test because both the sensors and the subjects can move freely in the space.

## Conclusion

In this report, an EEG electrode positioning method using photogrammetry is presented. By combining CCD and TOF cameras, the system can achieve both good accuracy (due to the precise industrial camera) and real-time efficiency (due to the reliable TOF camera). The vision system can reliably get the position and colors of the electrodes at the same time. A depth calibration plate for the TOF camera is designed, according to its distance-sensitive feature. Meanwhile, in order to improve the accuracy we apply the point cloud data to replace the traditional depth map with the calibration. In the experiments, we use a head model and 30-channel EEG electrode cap. The calibration process can be performed off-line, and the on-line acquisition algorithm can be realized in real-time, which can bring great convenience for patients and doctors. Thus, the combination of the TOF camera and the CCD camera can not only ensure the accuracy of positioning, but also simplify the complexity of the algorithm and operation.

## Author Contributions

SC: idea and conception; YH, HQ, and MZ: development of methods; HQ: experiments; HQ and XY: data analysis and programming; HQ: drafting the manuscript; SC, YH, XY, and MZ: critical revision.

### Conflict of Interest Statement

The authors declare that the research was conducted in the absence of any commercial or financial relationships that could be construed as a potential conflict of interest.
